# Effect of Culture Conditions on Fatty Acid Profiles of Bacteria and Lipopolysaccharides of the Genus *Pseudomonas*—GC-MS Analysis on Ionic Liquid-Based Column

**DOI:** 10.3390/molecules27206930

**Published:** 2022-10-15

**Authors:** Emerencia Mező, Fruzsina Hartmann-Balogh, Ibolya Madarászné Horváth, Anita Bufa, Tamás Marosvölgyi, Béla Kocsis, Lilla Makszin

**Affiliations:** 1 Institute of Bioanalysis, Medical School, Szentágothai Research Center, University of Pécs, 7622 Pécs, Hungary; 2 Department of Medical Microbiology and Immunology, Medical School, University of Pécs, 7622 Pécs, Hungary

**Keywords:** fatty acid profile, bacteria, lipopolysaccharides, cultivation conditions, gas chromatography-mass spectrometry, *Pseudomonas* genus, ionic liquid column

## Abstract

The profiling of bacterial fatty acids is a well-established technique in identifying and classifying bacteria. Cultivation conditions may affect the biosynthesis, thereby, changing the fatty acid profile in bacteria. The effect of the culture conditions on the fatty acid components of *Pseudomonas aeruginosa* PAO1, *Pseudomonas aeruginosa ATCC 27853*, *Pseudomonas aeruginosa* polyresistant and *Pseudomonas putida* all are aligned to the genus *Pseudomonas*. The fatty acids in the lipopolysaccharides of *Pseudomonas aeruginosa* PAO1 were also examined. The effects of the cultivation conditions were followed by using agar and blood agar media at the characteristic temperatures, 25 °C, 37 °C and 42 °C, respectively, and an analysis was made during the 1st, 3rd and 5th day following inoculation. In addition to quantitative differences, we also experienced qualitative differences in the fatty acid profiles which detect newly appearing fatty acids, due to changes in environmental factors. The application of ionic liquid-based column unveils new possibilities for the analyses of fatty acids in GC-MS experiments for bacterial fatty acid profiling. The validation results (response linearity, limit of detection, limit of quantification, system suitability, intraday and interday repeatability and accuracy) show the high separation efficiency of the ionic liquid-based column in the analyses.

## 1. Introduction

*Pseudomonas* is one of the most studied, medically important bacterial genera, since their species have a broad repercussion in public health. *P. aeruginosa* and *P. putida* are Gram-negative pathogenic bacteria, which are members of the fluorescent *Pseudomonas* strains. *P. aeruginosa* have become the most common bacterium causing severe respiratory and urinary tract nosocomial infections. One of the most common infections of *P. putida* is the catheter-associated bacteremia, whereas other infections include cholangitis, cholecystitis, pneumonia, or urinary tract infections.

The profiling of bacterial fatty acids (FAs) is a well-established technique for identifying and classifying bacteria. Fatty acids are mainly found in the cytoplasmic membrane as components of phospholipids and glycolipids, including in the lipoteic acid components of the cell wall of Gram-positive bacteria and in the lipopolysaccharide region of the outer membrane of Gram-negative bacteria. The outer surface of Gram-negative bacteria is covered with endotoxins, i.e., lipopolysaccharides (LPSs) or lipooligosaccharides (LOSs) [1]. This exterior protects the bacterium from antimicrobial effects and plays a decisive role in host adhesion and infectivity. Endotoxins have a dual biological effect. On the one hand, they are general stimulants of the immune system, and on the other hand, the immune system can become overloaded if they enter the bloodstream in larger quantities causing tissue damage, even death.

Qualitative and quantitative fatty acid analysis by gas chromatography has been an effective tool for distinguishing microorganisms for nearly sixty years. [2]. The total fatty acid bacterial profile of the cell directly and accurately reflects the cell genome. Bacterial fatty acids are, therefore, regularly used for taxonomic classification of bacteria including identification purposes [3]. The bacterial endotoxins are composed of fatty acids and carbohydrate chains, and the toxic activity is determined by the type and amount of the fatty acids. Classification of fatty acids can be made according to the structure, i.e., saturated fatty acids (SAFAs), monounsaturated fatty acids (MUFAs), polyunsaturated fatty acids (PUFAs), branched fatty acids (iso and anteiso forms), hydroxy fatty acids and cyclic fatty acids (e.g., 8-(2-octylcyclopropyl)octanoic acid). It is also possible to differentiate the fatty acids according to the number of the carbon atoms, i.e., short-chain fatty acids (SCFAs) containing fewer than 6 carbon atoms, medium-chain fatty acids (MCFAs) containing 7–12 carbon atoms, long-chain fatty acids (LCFAs) containing 13–21 carbon atoms, and very long-chain fatty acids (VLCFAs) containing 22–24 carbon atoms. 

Environmental factors, e.g., temperature and culture media, may significantly influence the biosynthesis of bacteria, of which, will affect the fatty acid composition. Membrane lipids and their associated fatty acids are suitable biomarkers for monitoring structural changes caused by external influences, since they are essential components of all living cells, with high structural diversity and high biological specificity. Several authors have shown the fatty acid content of the bacterial cell membrane can be altered in response to changes in environmental conditions, such as temperature [4], pH [5], age of the culture at harvest [6] or the presence of compounds in the culture medium [7]. Numerous attempts have been made with the purpose of revealing the relationship between the modification of fatty acid composition and its physiological relevance. It is now suggested the fatty acid composition affects the fluidity, flexibility and the permeability of the cell membrane. Zhang and Rock [8] have commented in detail regarding this phenomenon and demonstrated straight chain saturated fatty acids (SAFAs) produce a membrane bilayer with high rigidity and low permeability. Furthermore, it was found the presence of cis-unsaturated fatty acids (cis-UFAs) results in higher permeability. The branched chain fatty acids affect membrane fluidity, e.g., the anteiso fatty acids create a more fluid membrane structure than iso fatty acids. Moreover, cyclopropane fatty acids reduce the permeability of the cell membranes [9], and polyunsaturated fatty acids (PUFAs) are important for maintaining membrane fluidity [10]. Segura et al. [11] pointed out the change in the ratio of the long-chain and short-chain fatty acids plays a role in the regulation of membrane fluidity under unfavorable conditions. It was found the higher proportion of long-chain fatty acids can hinder the penetration of pollutants/toxic compounds/antibiotics into the membrane. The conversion of cis unsaturated fatty acids into their corresponding trans configuration was also demonstrated as a response to environmental changes which leads to a quick rigidification of the cell membrane [12]. Different environmental stresses can also induce changes in the bacterial endotoxins, including the formation of the biofilms [12]. Comparing *P. aeruginosa* biofilms with batch plankton cells, Chao et al. [13] showed how the ratio of each fatty acid class affects the properties of the cell membrane. In contrast to other studies, a less rigid membrane structure was observed due to a decrease in SAFA content and a decrease in the fatty acid chain length of the biofilms.

Studies on FAs and their metabolism are important in numerous research fields, including biology, bacteriology, ecology, human nutrition and health. Specific FAs and their ratios in cellular membranes may be used as biomarkers to enable the identification of organisms, to study adaptation of bacterial cells to toxic compounds and environmental conditions. The aim of this work is to monitor the changes in the fatty acid composition of bacteria belonging to the *Pseudomonas* genus due to external influences. The fatty acid compositions of bacteria cultured in different media, at different temperatures for short or longer incubation times were investigated by gas chromatography-mass spectrometry (GC-MS) using a separation column with an extremely polar ionic liquid stationary phase. In our previous work the ionic liquid based column compared by other capillary columns with different polarities was found as an accurate and suitable analysis for determination and quantification of FAME components [14].

## 2. Results

### 2.1. Separation on Ionic Liquid-Based Column

**Figure 1:**
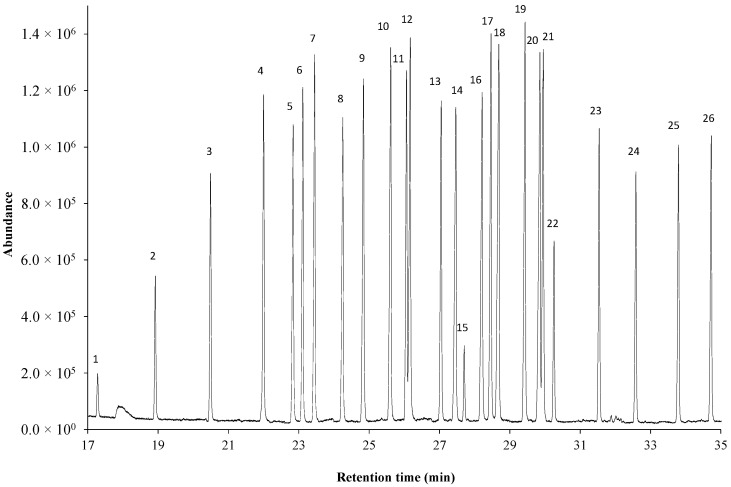
Total ion GC-MS chromatogram of BAME CP Mixture using the ionic liquid-based SLB-IL111 column. The peak labels represent components listed in Table 1 according to the retention order. The optimized experimental conditions are given in Section 4. The total FAME concentration is 0.5 mg mL^−1^.

Fatty acids were analyzed in methyl ester forms by GC–MS. The total ion chromatograms were registered using an ionic liquid (IL)-based column with extremely high polarity (SLB-IL111, 60 m). The analytical method was validated by applying a 26-component-fatty-acid-methyl-ester mixture (Bacterial Acid Methyl Esters CP Mixture—BAME CP Mixture) as described in the Section 4. The separated methyl ester components were identified by their mass spectra, and Table 1 contains the list of the components by the order of the retention times. Figure 1 shows the total ion chromatogram of the mixture.

The application of the SLB-IL111 column for the separation of the 26 fatty acid methyl-ester components including saturated, monounsaturated (cis, trans), polyunsaturated, branched (iso, anteiso), hydroxy and cyclopropane fatty acids provided baseline resolution with the exception in two cases (component 11–12 and component 20–21). In the latter cases, a slight overlaps regarding the peaks (R_11,12_ = 1.21; R_20,21_ = 1.05) were obtained. The optimized and validated separation method demonstrated the saturated fatty acid methyl ester components preceded the unsaturated ones, and the hydroxyl components are eluted long after the corresponding saturated fatty acid methyl ester. The cis monosaturated isomer of the octadecanoate component follows the saturated and the trans monosaturated isomer, while the octadecadienoate (C18:2cc) component follows all the other C18 isomers of this standard mix.

### 2.2. Evaluation of the Chromatographic Method

The chromatographic method was validated with the components of this BAME CP Mixture by determining the calibration curve equations, the coefficient of determinations (R^2^) and additional validation data (intraday and interday repeatability, system suitability, and average accuracy). Table S1 summarizes the data in detail (see Supplementary Materials).

The calibration curves were used to characterize the linear response for the determination of the fatty acid methyl esters, and the values of R^2^ were higher than 0.95 for the peak areas (concentration). The system suitability was characterized with RSD of the retention time (found between 0.01% and 0.03%) and of the concentration (found between 4.08% and 9.7%), which are below the generally accepted criteria (<2% and <10%, respectively). The RSD values obtained for the intraday and interday repeatability experiments were between 2.15% and 7.15% (the general criteria is <10%), and the RSD values for average accuracy were between 91.9% and 104.7% (values between 80% and 120% are acceptable). The LOD values for the components were between 0.03 and 0.27 µg mL^−1^, whereas the LOQ values were between 0.11 and 0.80 µg mL^−1^. The LOD and LOQ values for the BAME CP Mixture are listed in Table S2.

### 2.3. Fatty Acid Profiles of Bacteria

The fatty acid profiles of the bacteria, *Pseudomonas aeruginosa* PAO1 (PAO1) bacteria and its lipopolysaccharides (PSAE PAO1 LPS), *Pseudomonas aeruginosa ATCC 27853* (PSAE ATCC 27853), *Pseudomonas putida (P. putida)*, and *Pseudomonas aeruginosa* polyresistant (PSAE PR) bacteria cultivated at 25, 37 and 42 °C degrees on agar culture media were examined. The fatty acids were extracted from the bacteria and analyzed after derivatization in the methyl-ester forms with GC-MS applying the SLB-IL111 column for the separation. The extraction and derivatization procedures are described in the Section 4.The fatty acid methyl ester components were identified using the MS spectra and the retention times employing the results with the BAME CP Mixture and implementing the GLC-463 Reference Standard mixture. The latter contains fatty acid methyl esters, which are not present in the BAME CP Mixture, yet appear in the bacterial samples. 

#### 2.3.1. Representation of Optimal Conditions

**Figure 2:**
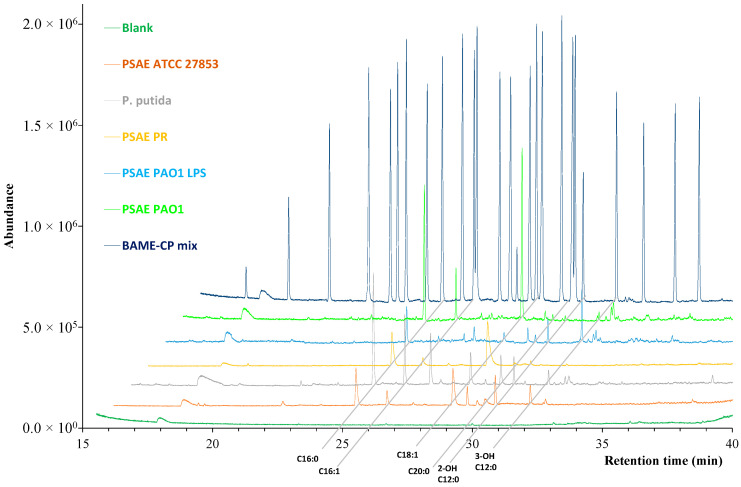
Total ion GC-MS chromatogram of *Pseudomonas* bacterial strains and PSAE PAO1 LPS, cultivated at 37 °C on agar cultivation media and on the first day of incubation, BAME CP Mixture and blank sample (the surface of the culture media without any bacteria) using the ionic liquid-based SLB-IL111 column. The main fatty acid components were indicated in the different samples. The optimized experimental conditions are given in Section 4.

Based on recommendations for in vitro cultivations of *P. aeruginosa* [15], the 37 °C growth temperature, the agar cultivation media and a one-day incubation time were considered as optimal conditions (Figure 2).

The greatest proportions of the fatty acids were the SAFAs, 60.6%, the MUFAs, 24.4% and the hydroxy fatty acids, 9.0% (see Table 2). The cyclopropane fatty acids (8.0%) were also detected in the isolates except in the case of *P. aeruginosa* polyresistant. Mainly LCFAs, and to a small extent MCFAs were present in the isolates under the ideal conditions. The VLCFAs were detected only in *P. aeruginosa* ATCC 27853, and SCFAs were not identified during the analyses.

As the growth temperature increased from 25 to 42 °C the concentration of straight chain SAFAs increased generally in the *Pseudomonas* spp. investigated, while those of the MUFAs decreased. Branched saturated fatty acids (in *iso* form) were only identifiable in the case of *P. putida* at 42 °C, while PUFAs were only detected in *P. aeruginosa* ATCC 27853 at 37 °C. The concentration of hydroxy fatty acids remained stable with temperature change. In consideration of the cyclopropane fatty acids, an initial increase followed with a decrease in the concentration were observed with increasing temperature. The proportion of LCFAs at all temperatures and for all bacteria were orders of magnitude and when compared, were higher to MCFAs.

SAFAs increased with the temperature in the cases of *P. aeruginosa* PAO1 and *P. putida*, while decreased in *P. aeruginosa* polyresistant. The content of C16:0 characteristic fatty acid was increasing in *P. aeruginosa* ATCC 27853, *P. aeruginosa* PAO1 and *P. aeruginosa* polyresistant, while in *P. putida* an increase between 25 and 37 °C and a decrease at 42 °C was observed. Generally, the proportion of C16:1c MUFA declined with an increase in temperature. The proportion of 2-OH C12:0 either showed an increase (*P. aeruginosa* PAO1), or a decrease followed by an initial increase (*P. aeruginosa* ATCC 27853). The concentration of 3-OH C12:0 remained nearly stable in *P. aeruginosa* PAO1 and *P. putida*, while in the case of *P. aeruginosa* ATCC 27853 at 37 °C represented the maximum abundance.

#### 2.3.2. Effect of Cultivation Temperature on the Fatty Acid Composition

**Figure 3:**
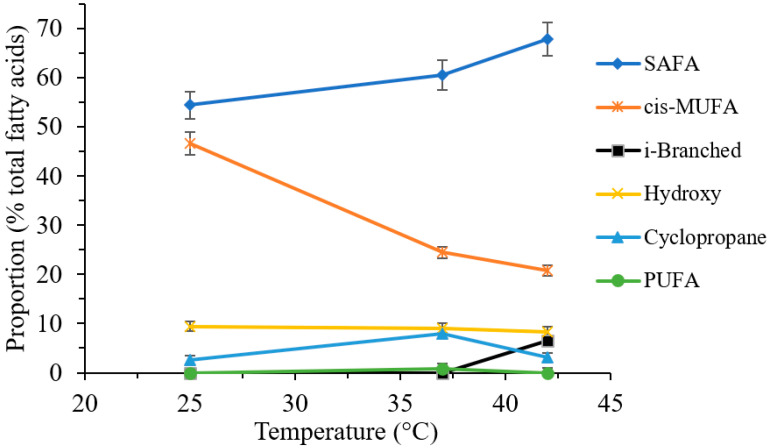
Influence of growth temperature on the mean proportion of fatty acid classes in bacteria (*P. aeruginosa* PAO1, *P. aeruginosa* ATCC 27853, *P. putida*, and *P. aeruginosa* polyresistant) cultivated at 25, 37 and 42 °C on agar cultivation media and on the first day of incubation (mean ± SD; *n* = 3). The optimized experimental conditions are described in Section 4.

The cultures were incubated at three varying temperature levels: 25 °C, 37 °C and 42 °C using agar culture media. Changes in the total fatty acid composition upon different cultivation temperatures for all bacterial isolates studied are listed in details in Table 3 and the mean changes in the proportion of distinguished fatty acid groups, as a function of temperature, is presented in Figure 3. 

Under these circumstances, 23 different fatty acids with 11 to 22 carbon atoms were detected in the five bacterial isolates of the genus *Pseudomonas*. All bacterial isolates contained 2-OH C12:0, C16:1c, C16:0 and C20:0 fatty acids (Table 3). The predominant fatty acid was the C19:0 in *P. aeruginosa* ATCC 27853 and in *P. aeruginosa* polyresistant, the C18:1 n-7 in *P. aeruginosa* PAO1, the 3-OH C12:0 in the lipopolysaccharide from *P. aeruginosa* PAO1, and the C16:0 in *P. putida*. 

Among the straight chain SAFAs, the C12:0, C14:0, C15:0, C16:0, C18:0, C19:0, C20:0, C22:0 fatty acids were identified. Only *P. aeruginosa* ATCC 27853 produced the C12:0 and C22:0 saturated fatty acids, although, the former was detected only in ~1% quantity. The C14:0 and C15:0 fatty acid were present only in *P. aeruginosa* ATCC 27853 and *P. putida*. While the C19:0 fatty acid was present only in *P. aeruginosa* ATCC 27853 and *P. aeruginosa* polyresistant. The C16:0 and C20:0 acids were produced by all studied bacteria. The C18:0 fatty acid was also found in all bacteria except in the case of *P. aeruginosa* polyresistant. Neither branched chain SAFAs (iso- and anteiso-isomers) fatty acids were found in the studied bacteria.

The cis monounsaturated fatty acid component, C16:1c is one of the characteristic fatty acids detected in all cases. C18:1c was present in *P. aeruginosa* PAO1 and its LPSs and in *P. putida*, within that the major component of the *P. aeruginosa* PAO1 with the proportion of 47.75. Trans MUFAs were not detected in any of bacterial isolates. 

2-OH C12:0 was identified in all bacterial isolates, whereas 3-OH C12:0 was absent in *P. aeruginosa* polyresistant. 2-OH C10:0 hydroxyl fatty acid was produced only by *P. aeruginosa* PAO1.

C17:0Δ and C19:0Δ cyclopropane fatty acids similarly absent in *P. aeruginosa* polyresistant and present in all the other isolates.

C20:2 n-6 was present alone in *P. aeruginosa* ATCC 27853 with a negligible, 0.8% abundance.

In comparing the *P. aeruginosa* PAO1 bacterium and its LPSs, it can be seen the major fatty acid of the bacteria was the C18:1c, while the most abundant fatty acid of the LPS was the 3-OH C12:0. Furthermore, LCFAs dominated in the bacteria, while MCFAs dominated in the LPS.

In *P. aeruginosa* PAO1 2-OH C10:0, C17:0Δ and C18:0 were synthesized when the temperature increased from 37 to 42 °C, while C20:0 was appeared only at 37 °C. In comparison, PAO1 LPSs formed C17:0Δ and C19:0Δ fatty acids only at 37 °C, in turn, C20:2 n-6 was present only at 42 °C. Furthermore, C20:0 fatty acid was synthesized when the temperature increased from 37 to 42 °C. *P. aeruginosa* ATCC 27853 formed new fatty acids C12:0, C14:0, C15:0, C18:0 and C20:2 n-6 at 37 °C. Additionally, C17:0Δ and C19:0Δ formed when the temperature increased from 37 to 42 °C. In *P. putida* C14:0, C15:0 and C19:0Δ present alone at 37 °C, while iC15:0, iC16:0, 2-OH C14:0, 3-OH C14:0 and C17:0 produced only at 42 °C. In *P. aeruginosa* polyresistant 2-OH C12:0 and C16:1c characteristic fatty acids formed when the temperature increased between 37 and 42 °C, while 3-OH C12:0 could be found only at 42 °C.

C11:0 fatty acid presents in a high abundance in *P. aeruginosa* PAO1 LPSs at 25 and 42 °C (33.6 and 46%) and interestingly, it disappeared at 37 °C. 2-OH C12:0, C16:1c and C17:0Δ fatty acids disappeared in *P. putida* as the temperature reached 42 °C. In *P. aeruginosa* polyresistant 2-OH C14:0, C17:0 and C18:0 was synthesized only at 25 °C, furthermore, C20:0 formation ceased at 42 °C. Similarly, C22:0 disappeared at 42 °C in *P. aeruginosa* ATCC 27853. 

The large quantities of LCFAs for all bacterial isolates remained unchanged in response to temperature. Notable change could be observed regarding PAO1 LPS, in which the MCFAs dominated at 25 and 42 °C. 

#### 2.3.3. Effect of Cultivation Media on the Fatty Acid Composition

The fatty acid composition of bacteria cultivated in agar was compared with commonly used blood agar media. The influence of applying different culture media are shown by the example of *P. aeruginosa* ATCC 27853 bacteria (Table 4).

Generally, SAFAs exhibited a greater proportion regarding blood agar medium at all temperatures, while hydroxy and cyclopropane fatty acids represented higher abundance in agar media. Additionally, a higher proportion of LCFAs was observed on blood agar medium, resulting in nearly 100% at all temperatures.

At 25 °C, the agar media showed greater proportion of C16:1c and C20:0, while in blood agar media twice the amount of C19:0 was indicated. In contrast, at 37 °C the blood agar media showed greater proportion of all three fatty acid components. At 37 °C, among the co-occurring fatty acids, 2-OH C12:0, 3-OH C12:0 and C19:0Δ were present in larger amounts on the agar medium, while C16:1c, C16:0, C19:0 and C20:0 showed a higher proportion on blood agar.

Interestingly, 2-OH C12:0 and 3-OH C12:0 hydroxy fatty acids disappeared at 25 °C in blood agar in comparison to agar media. Furthermore, C22:0 fatty acid was not pro-duced in blood agar media at 25 and 37 °C, as well as C12:0, C14:0, C15:0, C18:0, C22:0, C17:0Δ saturated fatty acids and C20:2 n-6 fatty acid disappeared at 37 °C in blood agar media. 

It was only in one case in which a new fatty acid formation in a response to changing cultivation media to blood agar was observed (C18:1c at 25 °C).

#### 2.3.4. Effect of Cultivation Media and Time on the Fatty Acid Composition

The effect of differences in experimental conditions were further studied with the comparison of fatty acid profiles regarding samples obtained from bacteria cultivated for one, three or five days on agar and blood agar media. Extraction and derivatization of fatty acids were made as described in the Materials and Method, and the optimized GC-MS method was applied for analysis using the SLB-IL111 column for separation. The results obtained in the case of *P. aeruginosa* ATCC 27853 bacteria are shown in Table 5.

**Figure 4:**
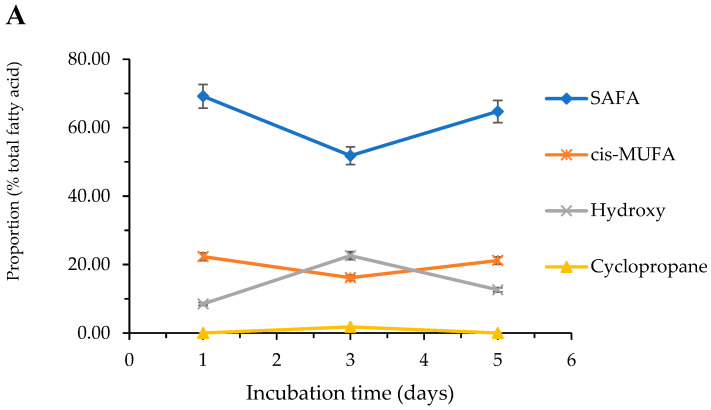

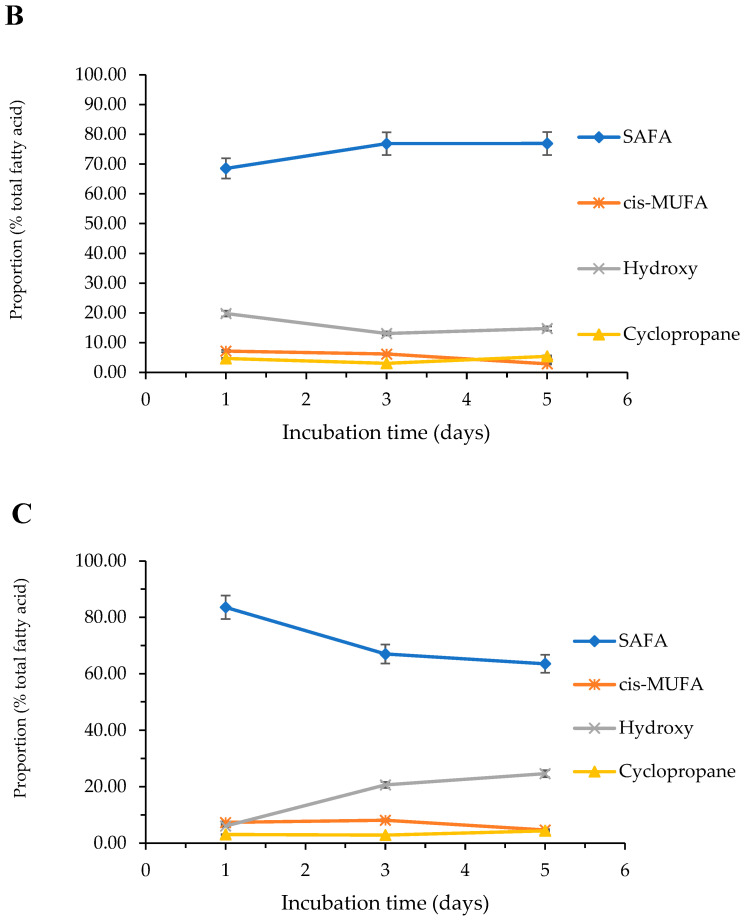
Influence of incubation time on the fatty acid proportion of *P. aeruginosa* ATCC 27853 grown at (**A**) 25 °C, (**B**) 37 °C and (**C**) 42 °C, using agar culture media (mean ± SD; *n* = 3). The optimized experimental conditions are detailed in Section 4.

In reference to agar media at 25 °C, the SAFAs and cis MUFAs declined between days 1 and 3, then increased between days 3 and 5, while the proportion of hydroxy and cyclopropane fatty acids appeared to initially increase up through the third day, then decreased until day 5 (Figure 4A). Many fatty acid components were detectable yet only on days 3 and 5 days at 25 °C, namely 2-OH C10:0, C14:0 and iC17:0.

At 37 °C, the amount of SAFAs gradually increased between days 1 and 3, then remained stable between days 3 and 5 (Figure 4B). The proportion of cis MUFAs gradually decreased over time, while total hydroxy and cyclopropane fatty acids declined between days 1 and 3, then increased between days 3 and 5.

At 42 °C SAFAs gradually declined, while hydroxy fatty acids gradually increased over time. Cis-MUFAs showed an increasing trend between days 1 and 3, then a decrease between days 3 and 5 (Figure 4C). Finally, cyclopropane fatty acids remained stable over time.

Generally, LCFAs dominated in the isolates with high ratios at all temperatures and days, yet only on the 3rd day, in which the proportion of MCFAs rose above 25%.

**Figure 5:**
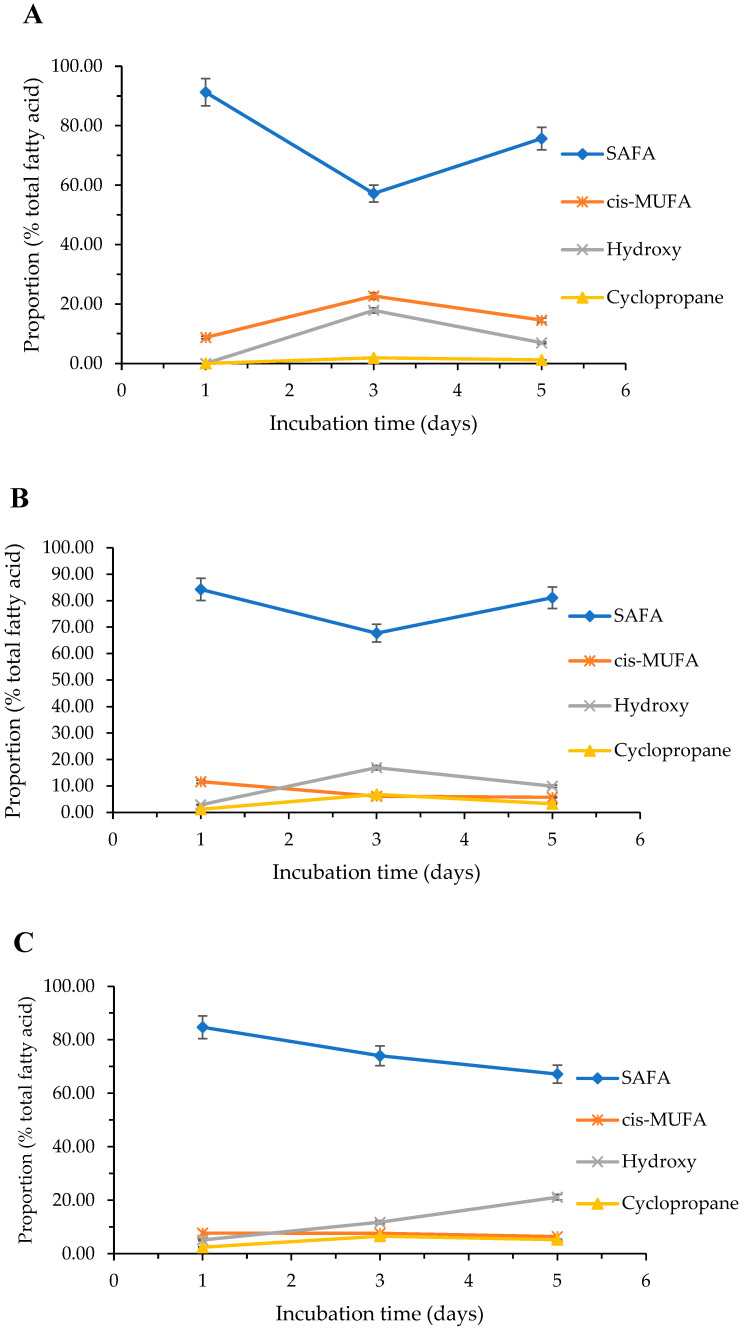
Influence of incubation time on the fatty acid proportion of *P. aeruginosa* ATCC 27853 grown at (**A**) 25 °C, (**B**) 37 °C and (**C**) 42 °C, using blood agar culture media (mean ± SD; *n* = 3). The optimized experimental conditions are detailed in Section 4.

In using blood agar media, similar trends in the proportion of fatty acid classes could be observed at 25 °C, in particular, using agar media (Figure 5A). SAFAs declined between days 1 and 3, then increased between days 3 and 5, while cis MUFAs, hydroxy and cyclopropane fatty acids appeared to increase between days 1 and 3, then showed a decrease between days 3 and 5. 

At 37 °C, SAFAs and cis-MUFAs indicated similar trends, in which their levels decreased initially up to days 3, then increased between days 3 and 5 (Figure 5B), while the abundance of hydroxy and cyclopropane fatty acids increased between days 1 and 3, then decreased between days 3 and 5.

At 42 °C, SAFAs and cis-MUFAs gradually decreased with the age of the culture, while as the cultivation time increased there were specific decreases in the levels of hydroxy fatty acids (Figure 5C). Finally, for the proportion of cyclopropane fatty acids, it increased between days 1 and 3, then decreased between days 3 and 5, as observed at previous temperatures.

Regarding the ratio of LCFAs and MCFAs, in general, there was a greater dominance of LCFAs on blood agar compared to agar, yet a similar trend could be observed in the proportions as the days progressed.

#### 2.3.5. Statistical Analysis

A large dataset was developed including the experimental parameters and the values obtained during the experiments. Different “analysis of variance” (ANOVA) procedures were performed towards discovering the relationship between the data. MANOVA was performed to determine a possible significant effect regarding the fixed factors on the dependent variables. MANOVA, including cultivation temperature (25; 37; 42 °C), cultivation medium type (agar, blood agar) and cultivation time (1, 3 and 5 days), as fixed factors showed statistically significant differences between the fatty acid components in *Pseudomonas aeruginosa* ATCC 27853: F(12) = 2.686, *p* = 0.002, Wilks’’ Λ = 0.818 and partial η2 = 0.065. Three-way ANOVA and two-way ANOVA were performed including several combination of independent variables. Statistically significant interaction on peak-area (F(4) = 4.198; *p* = 0.003 and partial η2 = 0.097) was obtained for the cultivation temperature × cultivation medium type × cultivation time variables, yet no significant interaction was obtained during the retention time (F(4) = 0.772; *p* = 0.545; partial η2 = 0.019) and area percentage (F(4) = 0.403; *p* = 0.806; partial η2 = 0.010). The two-way ANOVA procedures showed the cultivation temperature × cultivation days had statistically significant interaction in the peak-area, yet no significant interaction regarding retention time and area percentage. The cultivation temperature × cultivation medium type variables did not have statistically significant interaction on retention time, peak-area and area percentage. Similarly, the cultivation medium type × cultivation days did not have statistically significant interaction on retention time, peak-area and area %. The one-way ANOVA showed the cultivation medium type and cultivation days have statistically significant effects regarding peak-area (*p* = 0.001 and *p* = 0.044, respectively).

## 3. Discussion

Environmental factors are known to affect the biosynthesis of bacteria. For instance, bacteria can appear differently on various host organisms, e.g., at room temperature on hospital devices, or in the human body at 37 °C, or in a bird body at 42 °C, which may also be potential targets for the bacterium.

In this article, the influence of the temperature, cultivation media and incubation time on the fatty acid composition of *Pseudomonas aeruginosa* PAO1 bacteria and its lipopolysaccharides, *Pseudomonas aeruginosa ATCC 27853, Pseudomonas putida* and *Pseudomonas aeruginosa* polyresistant bacteria were studied by GC-MS using an extremely polar ionic liquid stationary phase. 

The results of the validation procedure indicate the method developed for the SLB-IL111 column is accurate, credible and suitable for the fatty acid methyl ester analyses. The system suitability, the intraday and interday repeatability and the average accuracy all corresponded to the recommended acceptance criteria (see in the Section 4). The wide variety of intermolecular interactions (i.e., dipole-dipole and hydrogen bonding interaction) provided by the ionic liquid stationary phase allowed the successful separation and the qualitative and quantitative determination of fatty acids of high structural heterogeneity from biological matrices, e.g., bacterial isolates. 

In the development of an infection, the first step is the colonization of the infective agent. In this process, the components of the bacterial cell surface play a basic role. They can bind the bacterial cells to the cytoplasmic membrane receptors of the host cells. The surface of *Pseudomonas* strains is rich in macromolecules much as in capsular polysaccharides, outer membrane proteins and lipopolysaccharides. These organic macromolecules possess a charge since they consist of different aminosugars, aminouronic and amino acids, including phosphate groups in their lipid-A part. Hydrophobic or hydrophilic character and charge of the bacterial surface are important in the attachment of bacterial cell to the target cell. This process is dependent upon both environmental conditions and the components of the bacterial cell wall. If the environmental conditions—temperature, pH or ion concentration—change, they can influence the biosynthetic processes in the bacterial cell. A change in the biosynthesis of macromolecules can influence their composition, their capability in the colonization and infective process. Analysis of the total fatty acids, which are present in high proportion in the membrane and which are characteristic for each bacterium, allows the monitoring of the effect of external influences.

Under optimal conditions (37 °C growth temperature, agar cultivation media and one-day incubation time), SAFAs, cis MUFAs, hydroxy and cyclopropane fatty acids were identified in the investigated *Pseudomonas* species in general (Table 2), which aptly concurs with previous results reported in the literature regarding the characteristic fatty acids of gram-negative bacteria [16]. Given that SAFAs were present generally in high proportions in the cultures indicates a rigid and less permeable membrane structure under optimal conditions [8]. This effect is further enhanced by the presence of a large amount (90%) of LCFAs [11].

A high abundance of cis-MUFAs was also observed, of which, these fatty acids are key precursors to cyclopropane and trans-MUFAs and maintain membrane fluidity and permeability [17]. 

Comparing the *P. aeruginosa* PAO1 bacteria and its LPS, significant differences were found mainly in the distribution of the components (Table 3). It is common knowledge, that hydroxy fatty acids are notable part of the LPS component of gram-negative bacteria [18]. Results show that while the bacterium is dominated by MUFAs, LPSs are dominated by hydroxy fatty acids. Generally, one of the main mechanisms of *P. aeruginosa* resistance to antibiotics is the maintenance of low permeability of the outer membrane [19]. This is reflected in the large amount of SAFAs and cyclopropane fatty acids which reduce membrane permeability and small amounts of cis-MUFAs, which will increase permeability. 

Studies have proven bacteria evolved including a number of mechanisms which al-low them to survive under adverse conditions. Since the first line of cell protection is based on the alteration of the membrane composition, of which, leads to lower fluidity and per-meability towards toxic compounds/pollutants/antibiotics, bacterial cells vary the fatty acid ratios of: (1) unsaturation to saturation, (2) cis to trans isomerization, (3) anteiso- to iso-branched structures, (4) short- to long-chain fatty acids, and (5) MUFAs to cyclopro-pane fatty acids. 

The comparison of the major fatty acid classes revealed the increased growth tem-perature from 25 to 42 °C resulted in changes regarding the distribution of saturated, un-saturated, branched, hydroxy and cyclopropane fatty acids. The increasing temperature changed the abundance of saturated fatty acids in all tested isolates. The observed in-crease in the abundance of SAFAs implies an increasing rigidity and a decreasing per-meability of the cell membrane, preventing antibiotics/pollutants to enter the cell. 

It has been shown in which cis-trans isomerization and the presence of trans-MUFAs enable *P. putida* to grow at higher temperatures, above the optimal 37 °C [20]. Although a decrease in the proportion of cis-MUFAs was observed in our experiments, contributing to a low fluidity and permeability membrane, the proportion of the corresponding trans-MUFAs did not increase with changes in temperature. However, increases in the ratio of cyclopropane fatty acids relative to their MUFA precursors were observed between the rise in temperature from 25 to 37 °C. Since the presence of cyclopropane fatty acids reduce the permeability of the cell membranes [9], it can be stated, that in our case, the membrane permeability and fluidity decreases to 37 °C.

Another observation was the appearance of branched fatty acids in response to temperature increase. Interestingly, i-C15:0, i-C16:0 and i-C17:0 could be detected in *P. putida* at 42 °C. In accordance with previous findings regarding the fatty acid composition of different *Pseudomonas* species [21], iso-branched fatty acids accounted for the total amount of branched fatty acids. Branched-chain fatty acids resist being packed very closely, thus making the membrane flexible, preventing it from becoming rigid [8], however, in such a small concentration (6.6%) as seen in our case, this effect was not prevalent.

Buchanan et al. demonstrated in which *P. aeruginosa* can modify the structure of its LPSs [22], i.e., the ratio of hydroxy fatty acids of different chain lengths. Their results can be linked to changes in antibiotic resistance of the bacterium. We found that among the detectable hydroxy fatty acids, 2-OH and 3-OH-C12:0 could be identified in PAO1 LPS, of which, the proportion changed in response to the increasing temperature. 

PUFAs are also essential for maintaining membrane fluidity [10]. Interestingly, these fatty acids did not appear in our assays, except for PAO1 LPS, when they appeared by ~20% at 42 °C.

Additional reactions to the changes in cultivation temperature were found to be the disappearance of fatty acids. Several fatty acids, mainly SAFAs, disappeared when the temperature was elevated to 37 or 42 °C. 

Both qualitative and quantitative differences were observed when comparing the fatty acid profiles obtained on agar and blood agar cultivation media. Despite their similarities, the fatty acid compositions in blood agar were significantly different from those obtained on conventional agar media. 

Some researchers addressed the comparison of traditionally used agar based culturing media regarding the fatty acid composition of bacteria. Walczak-Skierska et al. suggest that the differences in the fatty acid profiles are mainly attributed to nutritional dissimilarities of the media [23], and a higher fatty acid content was observed in the nutrient-rich medium. In spite of blood agar are enriched medium, in our experiments the conspicuous difference was the disappearance of fatty acids compared to the agar medium. In general, more fatty acids could be identified, and in many cases more intense peaks were observed on agar medium. 

Additionally, a notable difference in which the growing media caused was due to the change in the proportions of fatty acids. The greater proportion of SAFAs, and the higher proportion of LCFAs indicates a more inflexible and less permeable membrane structure in blood agar media. Scherer et al. [24] have compared the growth of *Helicobacter pylori* on fatty acid-free agar and on 5% sheep blood agar which contained fatty acids and differences in fatty acid profiles were attributed to a fatty acid uptake from the nutrient-rich medium. They have found saturated fatty acids are produced by the cells, thus, their presence in larger amounts on blood agar indicates SAFAs are additionally taken up from the blood agar growth medium. 

In only one case, specifically, a new fatty acid formation, C18:1c, occurred in blood agar medium, which is consistent with the findings of Scherer et al., who also proved the appearance of C18:1c is a consequence of uptake from the 18:1c-containing blood agar growth medium.

Incubation time also is found to have a notable effect on the concentration of fatty acids, using agar or blood agar medium. In general, the growth of bacteria follows an exponential distribution over time and can be modeled in four phases, the lag phase, exponential phase, stationary phase, and death phase [25]. The duration of the individual phases may differ by species. Studies on growth curves of *Pseudomonas* spp. have primarily examined the change in growth over maximum of 50 h [26,27], when the growth phase slowly reaches the death phase and bacteria die due to lack of nutrients. However, Al-kafaween et al. [28] demonstrated day three is the optimum cultivation period for *P. aeruginosa* to form a strong biofilm. Distinctively, we experienced the most intense fatty acid peaks on the 3rd day of incubation. 

Since the incubation time increased from day 1 to day 5, we noted mainly distribu-tional differences in the fatty acid profiles, yet in some cases, new fatty acids appeared on days 3 and 5 compared to day 1. Iso-branched fatty acids appeared on 3rd and 5th days in-dicating an increasing membrane fluidity which facilitates compounds to enter the cell membrane.

The 42 °C and the 5-day incubation time showed as large as a deviation from the ide-al conditions which caused an overall decrease in fatty acid abundances, except hydroxy fatty acids. Additionally, they showed an increasing tendency as the days progressed in agar and blood agar medium. This can be explained by the large amounts of polyhydroxyalkanoate (PHA) found to be accumulated in bacteria when nutrients are limited [29,30]. PHAs are composed of hydroxy fatty acids and are important carbon and energy storage compounds synthesized by various bacteria. 

Similarly, a significant decrease in the percentage composition of LCFAs was observed on the 5th day at 42 °C. However, regardless of the cultivation conditions, LCFAs dominated during the analyses. 

Gram-negative bacteria also have been shown to respond to limited nutrients by converting MUFAs to cyclopropane fatty acids [31]. Our results confirm this effect, namely in agar media, in which proportions of MUFAs are decreased, the amounts of cyclopropane fatty acids increased (e.g., at 25 °C between 1 and 3 days, and at 37 and 42 °C between 3 and 5 days). 

Although the fatty acid profiles of Pseudomonas spp. under diverse conditions have been described comprehensively, this kind of comparison of the bacteria belonging to the same genus, in addition, this kind of purpose in using the SLB-IL111 column with the de-tailed method development and validation procedure has not been previously described. Bacterium influences the permeability of the membrane, as well as its resistance to anti-microbial agents, by its structural changes due to external influences. Changes in the structure of bacteria can help reveal several unknown aspects of bacterial pathogenicity. The obtained results may create a new opportunity to reduce the virulence of pathogenic bacteria with the ability to cause disease.

## 4. Materials and Methods

### 4.1. Chemicals

The Bacterial Acid Methyl Esters CP Mixture (Matreya LLC., State College, PA, USA), a 26-component BAME mixture with chain-lengths from C11 to C20, containing saturated, unsaturated, hydroxy-, branched and cyclic-fatty acids, was used to validate a GC-MS analysis. The 10 mg mL^−1^ stock solution in methyl caproate was diluted ten times with n-hexane. The proportion is 0.043%, for each component. To ensure the detectability of hydroxyl-fatty acids on the ionic liquid-based column, acetylation was performed as described previously [32]. Briefly, the hexane phase was evaporated with a nitrogen stream and a mixture of pyridine (100 µL) and acetic anhydride (100 µL) was used at 100 °C (1 h) for acetylation. After evaporating the reaction mixture, a 1 mg mL^−1^ final solution was prepared using acetone. The solutions were stored at −20 °C prior to analysis. The GC-grade solvent n-hexane, acetone, pyridine and acetic anhydride were purchased from Merck Chemical Co (Merck, KGaA, Darmstadt, Germany). Some fatty acids were identified with the application of the GLC-463 Reference Standard (Nu-Chek-Prep Inc., Elysian, MN, USA).

### 4.2. Samples and Derivative Preparation

#### 4.2.1. Bacterial Strains

*Pseudomonas aeruginosa* PAO1, *Pseudomonas aeruginosa ATCC 27853, Pseudomonas putida* and *Pseudomonas aeruginosa* polyresistant bacteria were used in the experiments. The bacteria are available from our own strain collection at the Department of Medical Microbiology and Immunology, Medical School, University of Pécs, Pécs, Hungary. The strains were plated on Müller-Hinton agar (Oxoid Ltd., Basingstoke, UK) and 5% sheep blood agar media and incubated for at least 1 day at 25 °C, 37 °C and 42 °C. Fatty acid composition of bacterial strains was determined following 1, 3 and 5 days of the incubation. The antimicrobial sensitivity of strains was detected using a filter paper disc method. We used the product of Oxoid Ltd., Basingstoke, UK and we fully adhered to protocol suggested by Oxoid firm. The *Pseudomonas aeruginosa* ATCC 27853 was sensitive to ciprofloxacin, ceftazidime and imipenem—cilastatin, the *Pseudomonas aeruginosa* polyresistant was resistent to the same antimicrobial drugs.

#### 4.2.2. Preparation of Lipopolysaccharides 

Endotoxins extracted from *Pseudomonas aeruginosa* PAO1 bacteria cultivated at 25 °C, 37 °C and 42 °C were analyzed. Following cultivation, the bacteria were centrifuged by 5000 g for 10 min at 4 °C. The sediments were washed twice at 4 °C in physiological saline solution (500 mL) and dried in acetone. The LPSs were isolated by the hot phenol-water extraction method [33]. The lipopolysaccharides were purified by dialysis and ultracentrifugation at 4 °C three times by 100,000 g for four hours and lyophilized.

#### 4.2.3. Derivatization Procedure

The fatty acid composition of Gram-negative bacteria and its LPSs were analyzed using the methyl ester method of Sharmili et al. [34] and MIDI Technical Note (Newark, NJ, USA, http://www.midi-inc.com, accessed on 3 September 2022) followed by acetylation procedure (see above). Briefly, bacteria and LPSs were saponified with 1 mL of 3.75 M NaOH in 50% aqueous methanol at 100 °C for 30 min and rapidly cooled to room temperature. Free fatty acids were derivatized to obtain methyl ester derivatives with 2.0 mL of 3.25 M HCl in a methanol solution at 80 °C for 10 min, and rapidly cooled to room temperature. The fatty acid methyl esters were extracted from the aqueous phase with 1.25 mL of hexane- methyl tert-butyl ether (l:l *v*:*v*) in the shaker-thermostat for 10 min, and the acidified lower phase was discarded; and the extract was washed and neutralized with 3.0 mL of a 0.3 N NaOH solution in shaker-thermostat for 5 min, centrifuge 2000× *g* rpm for 5 min. The organic extract in the upper phase was the final FAME extract.

### 4.3. Gas Chromatography—Mass Spectrometry Analysis

An Agilent Technologies 6890N gas chromatograph with a 5975 mass selective detector (Agilent, Waldbronn, Germany) was used for the analysis of the fatty acid methyl ester components in the samples. The chromatograph and the detector conditions were as follows: flow-rate of the helium carrier gas, 1.5 mL min^−1^; injection mode, splitless; temperature of the injector, ion source, quadrupole mass analyzer, and transfer line 250 °C, 230 °C, 150 °C, and 260 °C respectively. The injection volume was 1 µL. The mass spectrometer was operated at 70 eV in the electron impact (EI) mode, and the scanned mass range was 50–400 amu. The following parameters of the GC-MS method were optimized. The separation profile, the mass spectra and the retention times were acquired by using the extremely polar column, SLB-IL111 (with phase composition, 1,5-di(2,3-dimethylimidazolium)pentane bis(trifluoromethylsulfonyl)imide; length, 60 m; inner diameter, 0.25 mm; film thickness, 0.20 µm; Sigma, St. Louis, MO, USA). Prior to the first use, the conditioning of the columns was carried out in full accordance to the factory recommendations, i.e., raising the temperature from 24 °C to 200 °C at the rate of 15 °C min^−1^, held at 200 °C for 30 min, then raised at the rate of 20 °C min^−1^ to a temperature that was 10 °C below the highest recommended value, and held for 120 min. 

To find the appropriate method for the separations, the method used in our previous work [14] to separate fatty acid methyl esters with high structural diversity and to test different stationary phase GC columns, served as a basis and was modified and the following settings were found to be suitable. The column temperature was initially held at 50 °C for 0 min, then raised to 220 °C at the rate of 4.5 °C min^−1^, held at 220 °C for 1 min, and raised to 260 °C at 50 °C min^−1^, held at final temperature for 2 min. Data analysis was performed using the GC/MSD CHEMSTATION (Version D.03.01, Agilent, Waldbronn, Germany) software. The fatty acids were identified with the help of the MS library (AMDIS Version 2.64, NIST; Freeware; http://chemdata.nist.gov/mass-spc/amdis, accessed on 25 November 2019).

### 4.4. Validation Procedure

The developed GC-MS method was subjected to validation based on the guidelines for the validation of chromatographic methods [35] and the acceptance criteria for the validation parameters were specified referring to international recommendations [36] and the work of Karnes and March [37]. Each standard sample was analyzed at least three times under the same conditions. Solution series of five (total) concentrations of the acetylated BAME CP Mix standard mixture (0.1, 0.25, 0.5, 0.75 and 1 mg mL^−1^ concentrations prepared in acetone) were analyzed. Calibration curves were established from three (*n* = 3) complete analyses under the same conditions. The limit of detection (LOD) and limit of quantification (LOQ) were determined as described in [38]. System suitability was expressed by the relative standard deviation (RSD) values of the retention time and the concentration obtained from seven (*n* = 7) complete analyses of each sample under the same conditions within one day. The general criteria for the system suitability were that the RSD values should be less than 2% for the retention time and less than 10% for the concentration. The precision of the methods was checked by intraday and interday experiments, as well. The intraday repeatability was obtained from three (*n* = 3) complete analyses of each sample under the same conditions within one day, and the interday repeatability was obtained from three (*n* = 3) complete analyses of each sample repeated on three consecutive days (*n* = 9). The general criteria for the intraday and interday repeatability were that the RSD values should be less than 10%. The mean values of repeatability were expressed by the RSD values. The general criteria for the average accuracy were that the RSD values should be between 80 and 120%.

### 4.5. Statistical Analysis

Retention time, time corrected area and area percentage of values were processed using the SPSS version 28.0 statistics software. Comparison of the means was achieved by multivariate analysis of variance (MANOVA) to determine those significant parameters (*p* < 0.05) which could differentiate the types of the ionic liquid. Cultivation temperature (25; 37; 42 °C), cultivation medium type (agar, blood agar) and cultivation time (1, 3, 5 days), were taken as the independent variables. Wilk’’s Lambda index was computed to determine a possible significant effect of the cultivation conditions regarding the retention time, time corrected area and area % of fatty acid components. 

One-way ANOVA was used for the comparison of the groups (cultivation temperature; cultivation days). The *p*-values less than 0.05 were considered to be significant.

## 5. Conclusions

This technique, including the complex sample derivatization procedure, and the validated GC-MS method, including the separation power of the ionic liquid column found to be adequate to investigate bacterial microorganisms and to monitor its structural changes by external influences. The fatty acid compositions markedly affected both quali-tatively and quantitatively by the conditions under which the cultures were grown. New fatty acids appeared or their proportions changed, while others disappeared as a result of changes in growth temperature, culture medium and incubation time. The ratio of the in-dividual fatty acids, and thus their change, plays a decisive role in the flexibility and per-meability of the bacterial cell membrane, which significantly affects the adhesion to the host cells and the effectiveness of antibiotic treatments. Furthermore, the changes also in-fluence the composition of the endotoxin, which can modify the intensity of the inflam-matory process in the body when it enters the body. Samples at 25 °C are significant in nosocomial infections, optimal for the plant pathogenic bacteria and, as this is the room temperature, it can help us to understand the hospital equipment mediated infections, while samples at 37 °C provide an idea of the structure of bacteria incubating in the human body. Culturing at 42 °C is well representative of the conditions among birds, optimal temperature for avian pathogens. 

## Figures and Tables

**Table 1:** Fatty acid methyl ester components of the BAME CP Mixture and their retention time values analyzed with GC-MS on an SLB-IL111 type 60 m long ionic liquid based separation column. The experimental conditions of the optimized method are given in the Section 4.

Peak Label	Compound ID	Name	Retention Time (Min) *
1	C11:0	Methyl undecanoate	17.28
2	C12:0	Methyl dodecanoate	18.92
3	C13:0	Methyl tridecanoate	20.49
4	C14:0	Methyl tetradecanoate	22.01
5	i C15:0	Methyl 13-methyltetradecanoate	22.84
6	ai C15:0	Methyl 12-methyltetradecanoate	23.12
7	C15:0	Methyl pentadecanoate	23.45
8	i C16:0	Methyl 14-methylpentadecanoate	24.26
9	C16:0	Methyl hexadecanoate	24.85
10	i C17:0	Methyl 15-methylhexadecanoate	25.62
11	C16:1c	Methyl hexadecenoate (cis-9)	26.07
12	C17:0	Methyl heptadecanoate	26.18
13	C17:0Δ	Methyl cis-9,10-methylenehexadecanoate	27.05
14	C18:0	Methyl octadecanoate	27.48
15	2-OH C10:0	Methyl 2-hydroxydecanoate	27.70
16	C18:1t	Methyl octadecenoate (trans-9)	28.22
17	C18:1c	Methyl octadecenoate (cis-9)	28.47
18	C19:0	Methyl nonadecanoate	28.70
19	C19:0Δ	Methyl cis-9,10-methyleneoctadecanoate	29.44
20	C20:0	Methyl eicosanoate	29.86
21	C18:2cc	Methyl octadecadienote (all cis 9,12)	29.96
22	2-OH C12:0	Methyl 2-hydroxydodecanoate	30.26
23	3-OH C12:0	Methyl 3-hydroxydodecanoate	31.55
24	2-OH C14:0	Methyl 2-hydroxytetradecanoate	32.59
25	3-OH C14:0	Methyl 3-hydroxytetradecanoate	33.80
26	2-OH C16:0	Methyl 2-hydroxyhexadecanoate	34.73

* RSD of the retention time was less than 0.2%.

**Table 2:** Fatty acid methyl ester presence in samples from *P. aeruginosa* PAO1, *P. aeruginosa* ATCC 27853, *P. putida*, and *P. aeruginosa* polyresistant bacteria based on the percentile peak area. The bacteria were cultivated at 37 °C on agar media. Fatty acids were extracted one day following inoculation. The relative standard deviation is less than 2%. Experiments were performed in triplicates.

Fatty Acids	PSAE PAO1	PSAE ATCC 27853	*P. putida*	PSAE PR	Mean
	% of total fatty acids				
Ʃ SAFA	36.1	68.6	46.1	91.5	60.6
Ʃ cis MUFA	55.9	7.2	28.2	6.4	24.4
Ʃ Hydroxy FA	4.3	19.8	10.6	2.1	9.0
Ʃ Cyclopropane FA	3.8	4.7	15.6	-	8.0
Ʃ PUFA	-	0.8	-	-	0.8
					
Ʃ MCFA	4.3	20.9	10.1	2.1	9.3
Ʃ LCFA	95.8	77.2	90.0	97.9	90.2
Ʃ VLCFA	-	3.0	-	-	0.7

**Table 3:** Fatty acid methyl ester presence in samples from *P. aeruginosa* PAO1, *P. aeruginosa* ATCC 27853, *P. putida*, and *P. aeruginosa* polyresistant bacteria based on the percentile peak area. The bacteria were cultivated at 25 °C, 37 °C and 42 °C on agar media. Fatty acids were extracted one day following inoculation. The relative standard deviation is less than 2%. Experiments are made in triplicates.

Fatty Acids	PSAE PAO1			PSAE PAO1 LPS			*P. putida*			PSAE PR			Retention Time (Min)
	25 °C	37 °C	42 °C	25 °C	37 °C	42 °C	25 °C	37 °C	42 °C	25 °C	37 °C	42 °C	
Saturated fatty acids (SAFAs)													
C11:0	-	-	-	33.6	-	46.0	-	-	-	-	-	-	17.28
C14:0	-	-	-	-	-	-	-	1.3	-	-	-	-	22.01
C15:0	-	-	-	-	-	-	-	0.7	-	-	-	-	23.45
C16:0	18.8	32.6	39.5	10.1	20.6	5.3	17.9	33.1	11.6	12.6	30.9	37.3	24.85
C17:0	-	-	-	-	-	-	-	-	3.8	10.1	-	-	26.18
C18:0	-	1.9	1.7	7.6	8.3	7.3	1.1	1.9	16.1	42.1	-	-	27.48
C19:0	-	-	-	-	-	-	-	-	-	19.0	57.0	46.0	28.70
C20:0	-	1.6	-	-	5.5	3.8	18.3	9.1	31.5	8.4	3.5	-	29.86
cis Monounsaturated fatty acids (MUFAs)													
C16:1c	26.7	11.1	8.3	4.9	2.8	3.7	35.1	18.0	-	-	6.4	10.6	26.07
C18:1c	47.4	44.7	39.9	7.4	6.3	7.5	8.3	10.2	16.6	-	-	-	28.47
Branched fatty acids													
iso													
i C15:0	-	-	-	-	-	-	-	-	3.2	-	-	-	22.84
i C16:0	-	-	-	-	-	-	-	-	2.6	-	-	-	24.26
i C17:0	-	-	-	-	-	-	-	-	0.7	-	-	-	25.62
Hydroxy fatty acids													
2-OH C10:0	-	1.2	0.8	-	-	-	-	-	-	-	-	-	27.70
2-OH C12:0	1.0	1.0	2.9	16.3	13.5	2.5	11.4	6.4	-	-	2.1	4.3	30.26
3-OH C12:0	3.2	2.0	3.9	20.0	29.6	4.1	5.7	3.7	4.6	-	-	1.8	31.55
2-OH C14:0	-	-	-	-	-	-	-	-	4.7	7.8	-	-	32.59
3-OH C14:0	-	-	-	-	-	-	-	-	4.3	-	-	-	33.80
Cyclopropane fatty acids													
C17:0Δ	-	1.3	1.3	-	4.7	-	2.3	14.9	-	-	-	-	27.05
C19:0Δ	2.8	2.5	1.7	-	8.7	-	-	0.6	-	-	-	-	29.44
Polyunsaturated fatty acids (PUFAs)													
C20:2cc	-	-	-	-	-	19.8	-	-	-	-	-	-	32.06
Ʃ SAFA	18.8	36.1	41.1	51.3	34.3	62.4	37.3	46.1	63.1	92.2	91.5	83.3	
Ʃ cis MUFA	74.1	55.9	48.3	12.3	9.1	11.2	43.3	28.2	16.6	-	6.4	10.6	
Ʃ Hydroxy FA	4.2	4.2	7.5	36.4	43.1	6.6	17.1	10.1	13.7	7.8	2.1	6.0	
Ʃ Cyclopropane FA	2.8	3.8	3.0	-	13.4	-	2.3	15.6	-	-	-	-	
Ʃ PUFA	-	-	-	-	-	19.8	-	-	-	-	-	-	
													
Ʃ MCFA	4.2	4.2	7.5	70.0	43.1	52.6	17.1	10.1	4.6	-	2.1	6.0	
Ʃ LCFA	95.8	95.8	92.5	30.0	56.9	47.4	82.9	89.9	95.4	100.0	97.9	94.0	

**Table 4:** Fatty acid proportions (% of total fatty acids) of *P. aeruginosa* ATCC 27853 bacteria grown at 25 °C, 37 °C and 42 °C using blood agar media. The fatty acid extraction was made in triplicates from cultivates on the 1st, the 3rd or the 5th day following inoculation. The relative standard deviation is less than 2%.

Fatty Acids	PSAE ATCC 27853		
	Blood Agar		
	25 °C	37 °C	42 °C
Saturated fatty acids (SAFAs)			
C16:0	12.7	27.7	26.8
C19:0	74.0	36.3	36.1
C20:0	4.6	20.2	21.8
cis Monounsaturated fatty acids (MUFAs)			
C16:1c	6.0	11.6	7.7
C18:1c	2.8	-	-
Hydroxy fatty acids			
2-OH C10:0	-	-	-
2-OH C12:0	-	1.9	3.2
3-OH C12:0	-	1.0	1.9
2-OH C14:0	-	-	-
3-OH C14:0	-	-	-
3-OH C16:0	-	-	-
Cyclopropane fatty acids			
C17:0Δ	-	-	1.0
C19:0Δ	-	1.2	1.5
			
Ʃ SAFA	91.3	84.3	84.7
Ʃ MUFA			
Ʃ cis	8.7	11.6	7.7
Ʃ Hydroxy FA	-	2.9	5.1
Ʃ Cyclic FA	-	1.2	2.4
			
Ʃ MCFA	-	2.9	5.1
Ʃ LCFA	100.0	97.1	94.9

**Table 5:** Fatty acid proportions (% of total fatty acids) of *P. aeruginosa* ATCC 27853 bacteria grown at 25 °C, 37 °C and 42 °C using agar and blood agar media. The fatty acid extraction was made in triplicates from cultivates on the 1st, the 3rd or the 5th day following inoculation. The relative standard deviation is less than 2%.

Fatty Acids	1 Day						3 Days						5 Days					
	Agar			Blood Agar			Agar			Blood Agar			Agar			Blood Agar		
	25 °C	37 °C	42 °C	25 °C	37 °C	42 °C	25 °C	37 °C	42 °C	25 °C	37 °C	42 °C	25 °C	37 °C	42 °C	25 °C	37 °C	42 °C
	(*n* = 3)																	
Saturated fatty acids (SAFAs)																		
C12:0	-	1.1	-	-	-	-	2.8	-	-	4.7								
C14:0	-	3.6	-	-	-	-	1.3	1.1	-	1.8	-	-	1.2	3.2	-	-	-	3.1
C15:0	-	0.8	-	-	-	-	0.4	1.1	-	1.0	-	-	-	-	-	-	-	-
C16:0	14.1	23.2	27.0	12.7	27.7	26.8	14.6	23.2	27.0	20.3	27.5	27.6	21.4	26.2	25.2	18.3	19.0	24.8
C17:0	-	-	-	-	-	-	-	-	-	-	-	-	-	1.7	-	-	-	-
C18:0	-	1.2	-	-	-	-	-	1.8	1.0	-	1.4	-	-	1.5	-	-	-	3.4
C19:0	39.7	28.3	36.7	74.0	36.3	36.1	25.8	30.9	26.0	24.8	28.1	29.8	42.2	26.6	24.1	54.6	27.5	22.5
C20:0	14.1	7.4	19.9	4.6	20.2	21.8	5.5	18.7	13.0	3.3	10.7	16.7	-	17.8	12.3	2.7	34.6	13.4
C22:0	1.3	3.0	-	-	-	-	1.5	-	-	1.3	-	-	-	-	2.7	-	-	-
Monounsaturated fatty acids (MUFAs)																		
cis																		
C16:1c	22.3	7.2	7.3	6.0	11.6	7.7	16.2	6.2	8.1	21.6	5.3	6.7	21.2	2.9	4.7	14.6	5.7	6.4
C18:1c	-	-	-	2.8	-	-	-	-	-	1.1	0.8	1.0	-	-	-	-	-	-
trans																		
C16:1t	-	-	-	-	-	-	5.1	-	-	0.4	2.4	-	-	-	-	1.7	-	-
Branched fatty acids																		
iso																		
i C17:0	-	-	-	-	-	-	2.5	-	-	-	-	-	2.6	-	-	-	-	-
Hydroxy fatty acids																		
2-OH C10:0	-	-	-	-	-	-	9.7	-	-	-	3.7	-	1.3	2.3	6.3	-	-	-
2-OH C12:0	6.3	11.6	4.4	-	1.9	3.2	9.3	6.3	12.1	13.8	8.8	9.1		6.3	7.9	3.4	5.2	13.7
3-OH C12:0	2.2	8.2	1.7	-	1.0	1.9	3.6	6.8	8.5	4.1	4.4	2.7	11.4	6.2	10.8	3.6	4.7	7.4
Cyclopropane fatty acids																		
C17:0Δ	-	1.6	1.2	-	-	1.0	0.9	0.8	1.4	1.1	2.4	3.3	-	2.0	1.4	-	-	2.1
C19:0Δ	-	3.1	1.8	-	1.2	1.5	0.9	2.2	1.4	0.8	4.5	3.2	-	3.4	3.0	1.2	3.2	3.1
Polyunsaturated fatty acids (PUFAs)																		
C20:2cc	-	0.8	-	-	-	-	-	0.8	1.4	-	-	-	-	-	1.7	-	-	-
Ʃ SAFA	69.2	68.6	83.6	91.3	84.3	84.7	51.8	76.9	67.0	57.2	67.7	74.1	64.7	76.9	64.3	75.7	81.1	67.2
Ʃ cis	22.3	7.2	7.3	8.7	11.6	7.7	16.2	6.2	8.1	22.7	6.1	7.6	21.2	2.9	4.7	14.6	5.7	6.4
Ʃ Branched FA																		
Ʃ iso	-	-	-	-	-	-	2.5	-	-	-	-	-	2.6	-	-	-	-	-
Ʃ Hydroxy FA	8.5	19.8	6.1	-	2.9	5.1	22.6	13.1	20.6	17.9	17.0	11.8	12.6	14.8	24.9	6.9	9.9	21.2
Ʃ Cyclopropane FA	-	4.7	3.0	-	1.2	2.4	1.8	3.1	2.9	1.9	6.8	6.5	-	5.4	4.4	1.2	3.2	5.2
Ʃ PUFA	-	0.8	-	-	-	-	-	0.8	1.4	-	-	-	-	-	1.7	-	-	-
																		
Ʃ MCFA	8.5	20.9	6.1	-	2.9	5.1	25.4	13.1	20.6	22.6	17.0	11.8	12.6	14.8	24.6	6.9	9.9	21.2
Ʃ LCFA	90.2	77.2	93.9	100.0	97.1	94.9	73.1	86.9	79.4	76.1	83.0	88.2	88.5	85.2	72.4	93.1	90.1	78.8
Ʃ VLCFA	1.3	3.0	-	-	-	-	1.5	-	-	1.3	-	-	-	-	2.7	-	-	-

## Data Availability

Not applicable.

## Supplementary Materials

The following supporting information can be downloaded at: https://www.mdpi.com/article/10.3390/molecules27206930/s1, Table S1: Calibration curve equations, coefficient of determinations, intra- and interday repeatability, system suitability and average accuracy percentage of the BAMEs, determined in the BAME CP Mixture standard mixture, Table S2: Limit of detection (LOD) and limit of quantification (LOQ) values for components in the BAME CP Mix determined by GC–MS using the optimized method for the SLB-IL111 GC column. The optimized experimental conditions are detailed in Section 4.

## Sample Availability

The bacteria and bacterial endotoxin samples are provided by the Department of Medical Microbiology and Immunology, Clinical Center, University of Pécs, Hungary.

## References

[1] Lukácová M., Barák I., Kazár J. (2008). Role of structural variations of polysaccharide antigens in the pathogenicity of Gram-negative bacteria. Clin. Microbiol. Infect..

[2] Abel K., Deschmertzing H., Peterson J.I. (1963). Classification of microorganisms by analysis of chemical composition I. Feasibility of utilizing gas chromatography. J. Bacteriol..

[3] Himelbloom B.H., Oliveira A.C.M., Shetty T.S. (2010). Rapid Methods for the Identification of Seafood Micro-Organisms.

[4] Kropinski A.M., Lewis V., Berry D. (1987). Effect of growth temperature on the lipids, outer membrane proteins, and lipopolysaccharides of *Pseudomonas aeruginosa* PAO. J. Bacteriol..

[5] Russell N.J., Evans R.I., ter Steeg P.F., Hellemons J., Verheul A., Abee T. (1995). Membranes as a target for stress adaptation. Int. J. Food Microbiol..

[6] Mrozik A., Labuzek S., Piotrowska-Seget Z. (2005). Changes in fatty acid composition in *Pseudomonas putida* and *Pseudomonas stutzeri* during naphthalene degradation. Microbiol. Res..

[7] Mrozik A., Piotrowska-Seget Z., Łabuzek S. (2004). Changes in whole cell-derived fatty acids induced by naphthalene in bacteria from genus *Pseudomonas*. Microbiol. Res..

[8] Zhang Y.M., Rock C.O. (2008). Membrane lipid homeostasis in bacteria. Nat. Rev. Microbiol..

[9] Cronan J.E., Luk T. (2022). Advances in the Structural Biology, Mechanism, and Physiology of Cyclopropane Fatty Acid Modifications of Bacterial Membranes. Microbiol. Mol. Biol. Rev..

[10] Washabau R.J., Day M.J. (2013). Chapter 32-Nutritional Strategies in Gastrointestinal Disease. Canine and Feline Gastroenterology.

[11] Segura A., Duque E., Mosqueda G., Ramos J.L., Junker F. (1999). Multiple responses of Gram-negative bacteria to organic solvents. Env. Microbiol..

[12] Eberlein C., Baumgarten T., Starke S., Heipieper H.J. (2018). Immediate response mechanisms of Gram-negative solvent-tolerant bacteria to cope with environmental stress: Cistrans isomerization of unsaturated fatty acids and outer membrane vesicle secretion. Appl. Microbiol. Biotechnol..

[13] Chao J., Wolfaardt G.M., Arts M.T. (2010). Characterization of *Pseudomonas aeruginosa* fatty acid profiles in biofilms and batch planktonic cultures. Can. J. Microbiol..

[14] Mező E., Bufa A., Páger C., Poór V., Marosvölgyi T., Kilár F., Makszin L. (2021). The Role of Ionic Liquid Interaction in the Separation of Fatty Acid Methyl Esters—Polyunsaturated Geometric Isomers in GC–MS. Separations.

[15] LaBauve A.E., Wargo M.J. (2012). Growth and laboratory maintenance of *Pseudomonas aeruginosa*. Curr. Protoc. Microbiol..

[16] Lingfa L. (2015). Study of membrane fatty acids of Gram-negative bacteria and its influence towards the terrestrial ecosystem. Res. J. Pharm. Biol. Chem. Sci..

[17] Kim B.H., Kim S., Kim H.G., Lee J., Lee I.S., Park Y.K. (2005). The formation of cyclopropane fatty acids in *Salmonella enterica serovar Typhimurium*. Microbiology.

[18] Lodowska J., Wolny D., Weglarz L., Dzierzewicz Z. (2007). The structural diversity of lipid A from Gram-negative bacteria. Postepy Hig Med. Dosw.

[19] Pang Z., Raudonis R., Glick B.R., Lin T.-J., Cheng Z. (2019). Antibiotic resistance in *Pseudomonas aeruginosa*: Mechanisms and alternative therapeutic strategies. Biotechnol. Adv..

[20] Junker F., Ramos J.L. (1999). Involvement of the cis/trans isomerase Cti in solvent resistance of *Pseudomonas putida* DOT-T1E. J. Bacteriol..

[21] Wayne Moss C., Dees S.B. (1975). Identification of microorganisms by gas chromatographic-mass spectrometric analysis of cellular fatty acids. J. Chromatogr. A.

[22] Buchanan P.J., Ernst R.K., Elborn J.S., Schock B. (2009). Role of CFTR, *Pseudomonas aeruginosa* and Toll-like receptors in cystic fibrosis lung inflammation. Biochem. Soc. Trans..

[23] Walczak-Skierska J., Złoch M., Pauter K., Pomastowski P., Buszewski B. (2020). Lipidomic analysis of lactic acid bacteria strains by matrix-assisted laser desorption/ionization time-of-flight mass spectrometry. J. Dairy Sci..

[24] Scherer C., Müller K.D., Rath P.M., Ansorg R.A. (2003). Influence of culture conditions on the fatty acid profiles of laboratory-adapted and freshly isolated strains of *Helicobacter pylori*. J. Clin. Microbiol..

[25] Paulton R.J.L. (1991). The bacterial growth curve. J. Biol. Educ..

[26] Khelissa S.O., Abdallah M., Jama C., Chihib N.E. (2019). Actively detached *Pseudomonas aeruginosa* biofilm cell susceptibility to benzalkonium chloride and associated resistance mechanism. Arch. Microbiol..

[27] Luo J., Kong J.L., Dong B.Y., Huang H., Wang K., Wu L.H., Hou C.C., Liang Y., Li B., Chen Y.Q. (2016). Baicalein attenuates the quorum sensing-controlled virulence factors of *Pseudomonas aeruginosa* and relieves the inflammatory response in *P. aeruginosa*-infected macrophages by downregulating the MAPK and NFκB signal-transduction pathways. Drug Des. Dev..

[28] Al-kafaween M.A., Mohd Hilmi A.B., Jaffar N., Al-Jamal H.A.N., Zahri M.K. (2019). Determination of optimum incubation time for formation of *Pseudomonas aeruginosa* and *Streptococcus pyogenes* biofilms in microtiter plate. Bull. Natl. Res. Cent..

[29] Madison L.L., Huisman G.W. (1999). Metabolic engineering of poly(3-hydroxyalkanoates): From DNA to plastic. Microbiol. Mol. Biol. Rev..

[30] Ayub N.D., Julia Pettinari M., Méndez B.S., López N.I. (2006). Impaired polyhydroxybutyrate biosynthesis from glucose in *Pseudomonas* sp. 14-3 is due to a defective beta-ketothiolase gene. FEMS Microbiol. Lett..

[31] Signature Lipid Biomarker (SLB) (2003). Analysis in Determining Changes in Community Structure of Soil Microorganisms. Pol. J. Environ. Stud..

[32] de Santana-Filho A.P., Noleto G.R., Gorin P.A.J., de Souza L.M., Iacomini M., Sassaki G.L. (2012). GC–MS detection and quantification of lipopolysaccharides in polysaccharides through 3-O-acetyl fatty acid methyl esters. Carbohydr. Polym..

[33] Westphal O., Jann K. (1965). Bacterial lipopolysaccharides. Extraction with phenol-water and further applications of the procedure. Methods Carbohydr. Chem..

[34] As S., Ramasamy P. (2016). Fatty Acid Methyl Ester (FAME) Analysis of Moderately Thermophilic Bacteria Isolated from the Coramandal Coast, Chennai, Tamilnadu. Eur. J. Exp. Biol..

[35] Taverniers I., De Loose M., Van Bockstaele E. (2004). Trends in quality in the analytical laboratory. II. Analytical method validation and quality assurance. TrAC Trends Anal. Chem..

[36] FDA Laboratory Manual Volume II: Methods, Method Verification and Validation. https://www.fda.gov/media/73920/download.

[37] Karnes H.T., March C. (1993). Precision, Accuracy, and Data Acceptance Criteria in Biopharmaceutical Analysis. Pharm. Res..

[38] Dodds E.D., McCoy M.R., Rea L.D., Kennish J.M. (2005). Gas chromatographic quantification of fatty acid methyl esters: Flame ionization detection vs. electron impact mass spectrometry. Lipids.

