# Supplementary material for: Effect of Culture Conditions on Fatty Acid Profiles of Bacteria and Lipopolysaccharides of the Genus Pseudomonas—GC-MS Analysis on Ionic Liquid-Based Column
Source: Molecules. 2022 Oct 15;27(20):6930. doi: 10.3390/molecules27206930 (PMC9610168; doi:10.3390/molecules27206930)
Supplement: Supplementary file 1 [file molecules-27-06930-s001.zip › molecules-1937093-supplementary.pdf]

Supplementary

Effect of culture conditions on fatty acid profiles of bacteria and lipopolysaccharides of the genus *Pseudomonas* – GC-MS analysis using an ionic liquid-based column

Emerencia Mező [1], Fruzsina Hartmann-Balogh [1], Ibolya Madarászné Horváth [1], Anita Bufa [1], Tamás Marosvölgyi[1], Béla Kocsis [2] and Lilla Makszin [1,*]

[1] Institute of Bioanalysis, Medical School, and Szentágothai Research Center, University of Pécs
[2] Department of Medical Microbiology and Immunology, Medical School, University of Pécs
* Correspondence: lilla.makszin@aok.pte.hu

Table S1 Calibration curve equations, coefficient of determinations, intra- and interday repeatability, system suitability and average accuracy percentage of the BAMEs, determined in the BAME CP Mix standard mixture.

Fatty acids	Calibration curve estimation[a]	Coefficient of determination (R^2)[b]	Repeatability		System suitability[e]		Average accuracy[f] (%)
			Intraday[c] (%RSD)	Interday[d] (%RSD)	Retention time (%RSD)	Concentration (%RSD)	
C11:0	y = 7.96E+06x + 1.08E+05	0.9951	4.53	3.81	0.03	4.25	100.9
C12:0	y = 2.61E+07x – 1.35E+05	0.9975	3.85	3.07	0.02	5.60	98.7
C13:0	y = 4.91E+07x – 8.95E+05	0.9923	2.15	2.21	0.02	5.68	99.5
C14:0	y = 6.65E+07x – 8.25E+05	0.9894	2.80	3.25	0.02	7.79	100.6
i C15:0	y = 7.20E+07x – 1.91E+06	0.9896	3.00	3.31	0.02	8.70	100.6
ai C15:0	y = 6.92E+07x – 1.71E+06	0.9946	2.69	2.50	0.02	9.70	100.5
C15:0	y = 7.66E+07x – 2.00E+06	0.9921	4.36	3.84	0.02	6.25	100.4
i C16:0	y = 7.87E+07x – 2.18E+06	0.9899	4.16	4.65	0.02	8.66	101.2
C16:0	y = 8.46E+07x + 5.15E+05	0.9929	4.23	3.78	0.02	5.59	99.9
i C17:0	y = 8.59E+07x – 1.56E+06	0.9994	2.14	2.74	0.02	5.28	99.5
C16:1c	y = 7.57E+07x – 1.39E+06	0.9975	2.41	3.76	0.01	5.81	99.8
C17:0	y = 8.60E+07x - 5.36E+05	0.9981	2.42	2.33	0.02	4.61	98.9
C17:0Δ	y = 7.85E+07x – 1.77E+06	0.9974	4.02	2.99	0.01	5.79	100.8
C18:0	y = 9.48E+07x – 2.86E+06	0.9975	3.64	2.67	0.02	9.68	101.0
2-OH C10:0	y = 1.49E+07x – 1.63E+05	0.9930	4.47	3.49	0.01	5.43	100.2
C18:1t	y = 9.06E+07x – 3.15E+06	0.9949	6.39	5.01	0.02	9.19	101.4
C18:1c	y = 8.91E+07x – 3.17E+06	0.9962	5.18	5.95	0.02	9.59	102.0
C19:0	y = 9.52E+07x – 2.25E+06	0.9626	6.43	6.26	0.02	6.39	99.6
C19:0Δ	y = 8.71E+07x – 2.28E+06	0.9991	5.14	6.05	0.02	9.02	100.4
C20:0	y = 1.21E+08x – 9.24E+06	0.9793	5.54	5.29	0.03	8.17	104.7
C18:2cc	y = 5.72E+07x + 4.51E+06	0.9511	4.48	3.20	0.01	5.54	91.9
2-OH C12:0	y = 4.11E+07x – 1.49E+06	0.9944	5.15	5.64	0.01	4.08	102.2
3-OH C12:0	y = 7.40E+07x – 2.42E+06	0.9975	7.15	6.35	0.02	4.12	101.0
2-OH C14:0	y = 5.73E+07x – 1.17E+06	0.9956	4.36	4.34	0.01	5.44	101.0
3-OH C14:0	y = 6.95E+07x – 3.63E+06	0.9964	4.30	4.95	0.02	5.72	101.3
2-OH C16:0	y = 6.63E+07x – 1.10E+06	0.9954	5.34	5.64	0.01	5.33	101.2

a. The calibration curve established from three (n = 3) complete analyses of five calibration solutions (0.1, 0.25, 0.5, 0.75 and 1 mg mL^{-1} concentrations prepared in acetone) in a day.
b. Calibration range was from 0.1 mg mL^{-1} to 1 mg mL^{-1}.
c. The value of %RSD of concentration established from three (n = 3) complete analyses of each sample in a day. The concentration was 0.75 mg mL^{-1}.
d. The value of %RSD of concentration established from three complete analyses repeated tree consecutive days (n = 9). The concentration was 0.75 mg mL^{-1}.
e. The value of %RSD of retention time and concentration established from seven (n = 7) complete analyses of each sample in a day. The concentration was 0.5 mg mL^{-1}.
f. Percentage of average accuracy of concentration established from three (n = 3) complete analyses of five calibration solutions in a day.

Table S2 Limit of detection (LOD) and limit of quantification (LOQ) values for components in the BAME CP Mix determined by GC–MS using the optimized method for the SLB-IL111 GC column. The optimized experimental conditions are detailed in Materials and Methods.

**Fatty acids**	**LOD (μg mL^{-1})[a]**	**LOQ (μg mL^{-1})[b]**
C11:0	0.08	0.25
C12:0	0.06	0.18
C13:0	0.10	0.31
C14:0	0.12	0.37
i C15:0	0.12	0.37
ai C15:0	0.09	0.26
C15:0	0.11	0.32
i C16:0	0.12	0.36
C16:0	0.10	0.30
i C17:0	0.03	0.09
C16:1c	0.06	0.18
C17:0	0.05	0.15
C17:0Δ	0.06	0.18
C18:0	0.06	0.18
2-OH C10:0	0.10	0.30
C18:1t	0.08	0.25
C18:1c	0.07	0.22
C19:0	0.07	0.20
C19:0Δ	0.04	0.11
C20:0	0.17	0.52
C18:2cc	0.27	0.80
2-OH C12:0	0.09	0.27
3-OH C12:0	0.06	0.18
2-OH C14:0	0.08	0.24
3-OH C14:0	0.07	0.21
2-OH C16:0	0.08	0.24

[a] The LOD values were measured at S/N ratio > 3.
[b] The LOQ values were measured at S/N ratio > 10.
